# Coordinated Temporal Convolutional Network and Transformer for enhanced carbon emission prediction in energy intensive industries

**DOI:** 10.1038/s41598-026-49249-w

**Published:** 2026-04-20

**Authors:** Bowen Zheng, Mingming Pan, Chang Liu, Jing Zhang, Jie Tong, Dezhi Li, Jianfeng Li, Zhong Zhuang, Meimei Duan, Kaijie Fang, Yixuan Huang, Jindou Yuan, Zixuan Guo, Yongjun Li

**Affiliations:** 1 China Electric Power Research Institute, Beijing, 100192 China; 2 State Grid Jiangsu Marketing Service Center, Nanjing, 210019 China

**Keywords:** Carbon emission prediction, Energy-intensive industries, TCN-Transformer, Steel industry, Concrete industry, Electrolytic aluminum, Electrical and electronic engineering, Energy grids and networks

## Abstract

This research presents a systematic framework for high-precision carbon emission forecasting in energy-intensive industries, integrating emission accounting methodologies with a Temporal Convolutional Network (TCN)-Transformer hybrid architecture. Motivated by the need for more robust short-term emission prediction, the study first establishes a theoretical foundation by comprehensively reviewing and categorizing prevalent carbon accounting approaches—including direct and indirect emission estimation based on the Intergovernmental Panel on Climate Change (IPCC) emission factor method—applied to representative enterprises in the steel, concrete, and electrolytic aluminum sectors. Building on this, a novel hybrid model is developed that combines the TCN’s ability to extract multi-scale temporal features using dilated convolutions with the Transformer’s capability to capture long-range dependencies through multi-head self-attention. Empirical validation on real-world datasets demonstrates that the proposed model significantly outperforms standalone Transformer architectures, yielding a 35% improvement in prediction accuracy and reducing the Mean Absolute Percentage Error (MAPE) to 3.21%. These results highlight the framework’s effectiveness in modeling complex emission dynamics, offering a scalable and data-driven tool to support proactive carbon management strategies in high-emission industrial contexts.

**Supplementary Information:**

The online version contains supplementary material available at 10.1038/s41598-026-49249-w.

## Introduction

In recent years, energy conservation and emission reduction have emerged as global imperatives, driven by growing concerns about climate change and increasingly stringent carbon policies. High-energy-consuming sectors—particularly steel, concrete, and electrolytic aluminum industries—have become focal points of decarbonization strategies due to their significant contributions to national greenhouse gas emissions. Statistical analyses reveal that these industries emit over 26,000 kilotons of CO₂-equivalent annually, with approximately 1,500 new key emission entities contributing to an additional 3 billion tons of emissions. This sharp increase imposes not only environmental burdens but also amplifies the complexity of power system operations, which must concurrently evolve toward low-carbon and renewable energy integration to support industrial emission reduction targets. Moreover, initial evaluations indicate that 15% of crude steel and 16% of concrete production capacity still fall short of energy efficiency benchmarks, revealing vast potential for emission abatement through operational optimization^1^. Innovative technologies and accurate forecasting are needed to effectively control high emissions^2^.

Accurate carbon emission forecasting serves as a cornerstone for implementing effective energy management strategies, emission trading systems, and real-time dispatch planning. Traditional statistical models, though interpretable, often fail to capture the nonlinear, high-dimensional temporal dependencies characteristic of industrial emission data. In contrast, deep learning techniques demonstrate superior performance by automatically learning complex representations from massive datasets and facilitating multi-task forecasting across temporal and spatial dimensions^3,4^.

Among deep learning models, Recurrent Neural Networks (RNNs) and their variants, such as Long Short-Term Memory (LSTM) and Gated Recurrent Unit (GRU), have proven effective in capturing sequential dependencies. LSTM mitigates issues like vanishing gradients through its memory-forgetting mechanism, enabling long-term dependency modeling^5^, while GRU provides a more computationally efficient alternative with comparable accuracy, particularly suited for short-term load forecasting tasks^6^. Despite these advances, the sequential nature of RNN-based architectures limits training parallelism and computational efficiency. To address this, the Transformer architecture, introduced by Google in 2017, revolutionized time series modeling with its self-attention mechanism, enabling parallel computation and significantly enhancing both training speed and inference throughput^7^.

Another promising approach is the Temporal Convolutional Network (TCN), a convolutional model specifically adapted for sequence modeling. By utilizing causal and dilated convolutions, TCNs expand receptive fields and preserve temporal order without recurrence, making them ideal for capturing long-range dependencies in emission data. Their ability to extract multi-scale temporal patterns has led to successful applications in load forecasting and spatio-temporal sequence analysis^8^. For instance, a hybrid model integrating TCN was proposed^9^, with Multihead Self-Attention, and BiLSTM to achieve superior accuracy in power load forecasting through hierarchical feature extraction and attention-enhanced generalization^10^.

The TCN-Transformer model, which combines Temporal Convolutional Networks (TCN) with Transformer architecture, has demonstrated notable advantages in modeling non-linear and non-stationary time series, offering strong capabilities in capturing both local temporal dependencies and long-range global patterns, thereby enabling high-precision carbon emission prediction. In contrast, the DCEF model integrates Empirical Mode Decomposition (EMD), Truncated Singular Value Decomposition (TsvD), and the ARIMA algorithm to achieve data stabilization, noise reduction, and linear time series forecasting. This composite approach enhances short-term forecasting efficiency but lacks the non-linear modeling flexibility of deep learning architectures^11^.

To address longer temporal dependencies and semantic-level alignment, the SSA-Attention-BiGRU model introduces a framework combining signal decomposition (SSA), bidirectional GRUs, and external attention mechanisms, which improves long-sequence forecasting for carbon neutrality trends but incurs higher computational complexity^12^. Expanding beyond structured data, a multimodal model based on ALBEF is proposed to integrate computer vision and natural language processing, enabling cross-modal understanding of carbon-related environmental issues; unlike TCN-Transformer, this model focuses on semantic interpretation rather than pure temporal prediction^13^. Moreover, for ensuring system robustness in carbon offsetting operations, the ResNet-BiGRU-TPA model combines convolutional feature extraction, bidirectional temporal modeling, and time-aware attention to effectively detect anomalies such as CO₂ leakage and system failures, offering strong performance in safety-critical scenarios^14^. Compared to these methods, TCN-Transformer provides superior scalability, faster training convergence, and stronger generalization across heterogeneous industrial data. The other models, while effective in specific scenarios, often suffer from limited adaptability to high-resolution enterprise-level forecasting tasks. This paper therefore focuses on leveraging the TCN-Transformer architecture to predict carbon emissions from energy-intensive enterprises, aiming to support fine-grained, enterprise-specific emission reduction strategies with high temporal resolution and modeling precision.

Motivated by these advancements, this study proposes a hybrid TCN-Transformer framework for high-resolution, short-term carbon emission forecasting in high-energy industrial enterprises. By integrating the temporal abstraction capability of TCN with the global dependency modeling strength of Transformer, the framework aims to overcome the limitations of individual models and offer a robust, scalable solution for emission pattern recognition and proactive carbon management:

1. This study systematically reviews and categorizes three prevalent carbon accounting methodologies employed in energy-intensive industries, establishing a theoretical framework for data-driven emission forecasting.
2. A novel hybrid architecture integrating TCN and Transformer models is proposed. The TCN component leverages dilated convolutions to capture multi-scale temporal patterns, while the Transformer module employs self-attention mechanisms to model long-range dependencies, thereby enhancing prediction accuracy.
3. Empirical validation on real-world industrial emission datasets demonstrates that the proposed model outperforms baseline methods, achieving a 35% improvement in prediction accuracy and reducing the MAPE to 3.21%.

## Methodologies for accounting of carbon emissions from energy-intensive industries

In this paper, the carbon emission calculation method for high-energy-consuming enterprises is based on the international standard ISO 14,064. It primarily divides the carbon emissions of these enterprises into two aspects: direct emissions and indirect emissions. Direct carbon emissions primarily comprise the total carbon emissions resulting from the combustion of fuels and processes in energy-intensive industries. Indirect carbon emissions mainly refer to the carbon emissions generated by electricity and heat consumed in the production process, in addition to supply chain emissions, i.e., emissions generated by transport and logistics. The core idea of calculating carbon emissions from both direct and indirect sources comes from Eq. (1): 

1 $$\:E=\sum\:_{i=1}^{n}{AD}_{i}\times\:{EF}_{i}$$

In Eq. (1), $\:E$ denotes the carbon emissions from a specific emission source, measured in tons of CO₂-equivalent. The term $\:{AD}_{i}$ represents the activity data corresponding to the emission source in kg/(kW⋅h) or GJ/t, such as the quantity of raw materials consumed, fuel usage, or process-level operational data. $\:{EF}_{i}$denotes the emission factor, which quantifies the amount of CO₂ released per unit of activity in kg CO_2_ per ton of material or kg CO_2_ per GJ of fuel, and is typically determined based on authoritative emission factor databases. In this study, the $\:{AD}_{i}$ values range from 10^4^ to 10^7^ units across industries, and $\:{EF}_{i}$ values are typically within the range of 0.1–3.2 kg CO_2_/unit.

This emission quantification methodology is applicable across both direct and indirect sources. By aggregating emissions from all relevant sources, the total carbon emissions of an enterprise can be calculated using the general formulation shown in Eq. (2).

2 $$\:{E}_{total}={E}_{fuel}+{E}_{produce}+{E}_{EH}+{E}_{transport}$$

In Eq. (2), the total carbon emissions $\:{E}_{total}$ of an enterprise, measured in tons of CO₂-equivalent, are decomposed into four major components: $\:{E}_{fuel}$ carbon emissions from fuel combustion; $\:{E}_{produce}$ emissions generated during industrial production processes; $\:{E}_{EH}$ indirect emissions resulting from the consumption of externally supplied electricity and thermal energy; and $\:{E}_{transport}$ emissions associated with the transportation of materials throughout the supply chain. Each component is expressed in tons and corresponds to a specific emission source, allowing for a structured and comprehensive accounting of the enterprise’s overall carbon footprint.

### Calculation of carbon emissions from steel companies

Accounting for carbon emissions from steel companies includes the calculation of carbon emissions from three components. Firstly, carbon emissions from the combustion of fossil fuels, secondly, carbon emissions from the production process, which together serve as the direct carbon emissions of the iron and steel enterprise, and lastly, indirect carbon emissions from the consumption of electrical and thermal energy. The total carbon emissions of the iron and steel enterprises can be expressed by Eq. (3):

3 $$\:{E}_{steel}={E}_{Fuel}+{E}_{produce}+{E}_{EH}$$

In Eq. (3), $\:{E}_{steel}$ represents the total carbon emissions of a given steel enterprise, measured in tons of CO₂-equivalent. The term $\:{E}_{Fuel}$corresponds to carbon emissions from fuel combustion, while $\:{E}_{produce}$ accounts for process-related emissions generated during the steel manufacturing stages. $\:{E}_{EH}$ refers to indirect emissions arising from the consumption of electricity and thermal energy. All components are quantified in tons, providing a comprehensive accounting framework for steel industry emissions.

**Fig. 1:**
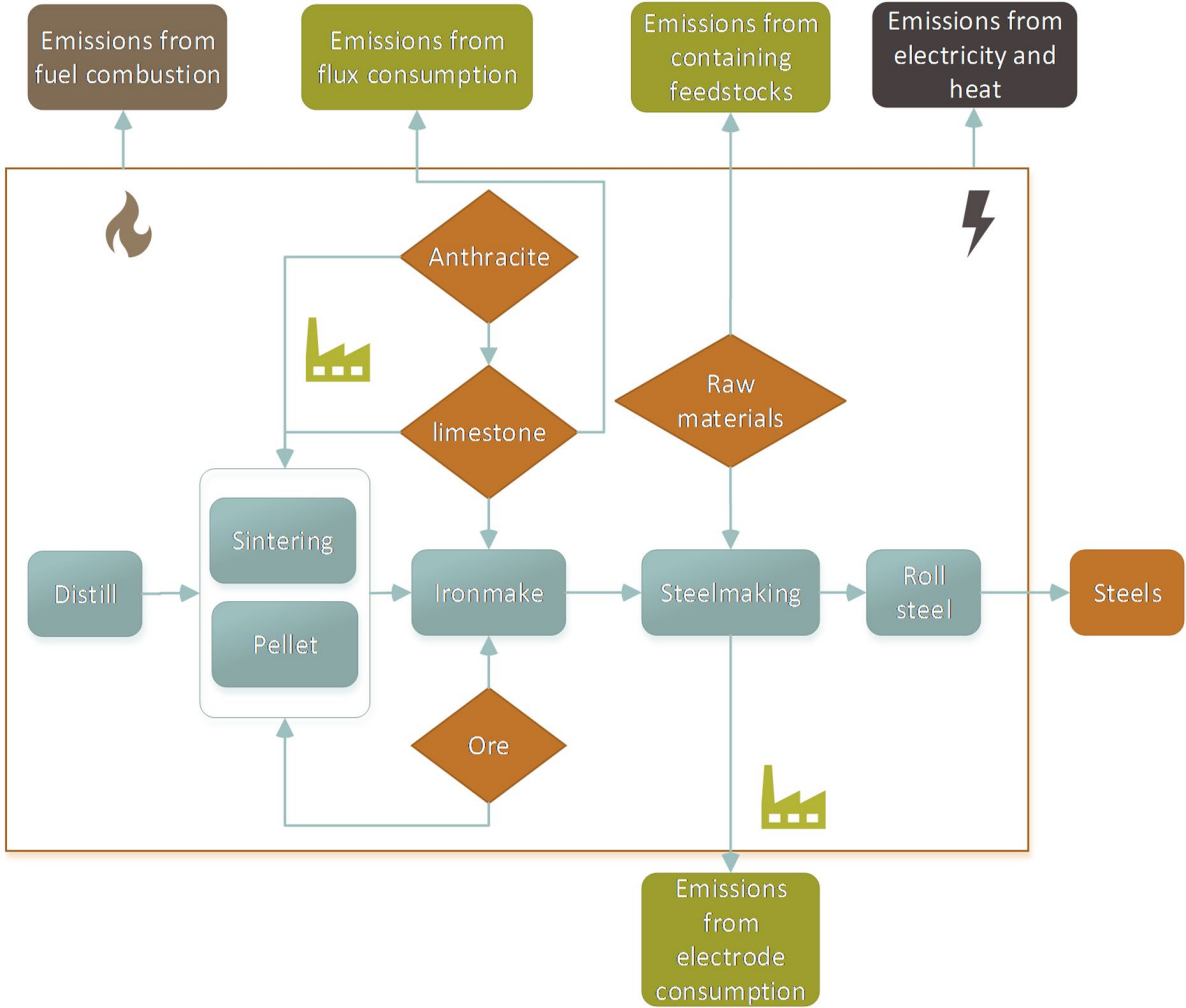
Carbon emission accounting boundary for steel enterprises.

In accordance with the ISO 14064 series of standards, the carbon emission accounting boundary for steel enterprises is clearly defined. As illustrated in Fig. 1, these boundaries distinguish between direct and indirect emission sources and guide the inclusion of relevant processes in carbon inventories.

The carbon emissions generated from fossil fuel combustion can be quantified using the calculation method defined in Eq. (4).

4 $$\:{E}_{Fuel}={\sum\:}_{i=1}^{n}{AD}_{i}\times\:{EF}_{i}$$

In Eq. (4), $\:{E}_{Fuel}$ denotes the carbon emissions from fuel combustion in a steel enterprise, measured in tons of CO₂-equivalent. The term $\:{AD}_{i}$ refers to the activity data related to fuel usage, which serves as an indicator of the scale or output level of steel production and is typically expressed in terms of the amount of various fossil fuels consumed. $\:{EF}_{i}$ represents the carbon emission factor associated with each specific type of fuel, indicating the amount of CO₂ emitted per unit of fuel consumed.

The estimation of carbon emissions in the steel production process is obtained by adding the carbon emissions from melt consumption, electrode consumption and consumption of carbon-containing raw materials in each step, which can be calculated by Eq. (5).

5 $$\:{E}_{produce}={E}_{Melt}+{E}_{Electrodes}+{E}_{Material}$$

Indirect CO2 emissions from electricity and heat consumption can be calculated using Eq. (6):

6 $$\:{E}_{EH}={AD}_{E}\times\:{EF}_{E}+{AD}_{H}\times\:{EF}_{H}$$

In Eq. (6), $\:{AD}_{E}$ and $\:{AD}_{H}$ represent the consumption of electricity and heat; $\:{EF}_{E}$ and$\:{\:EF}_{H}$ represent the carbon emission factors for electricity and heat, respectively.

### Calculation of carbon emissions from concrete processes

Carbon emission accounting for concrete generally starts from a particular project and includes the calculation of carbon emissions for both direct and indirect components. That is, direct carbon emissions from the combustion of fossil fuels and the production of raw materials, and indirect carbon emissions from the consumption (e.g., electricity, water resources, etc.) and transport of intermediate parts of the project construction process. The total carbon emissions of a concrete project can be expressed in Eq. (7):

7 $$\:{E}_{concrete}={E}_{fuel}+{E}_{produce}+{E}_{EW}+{E}_{transport}$$

**Fig. 2:**
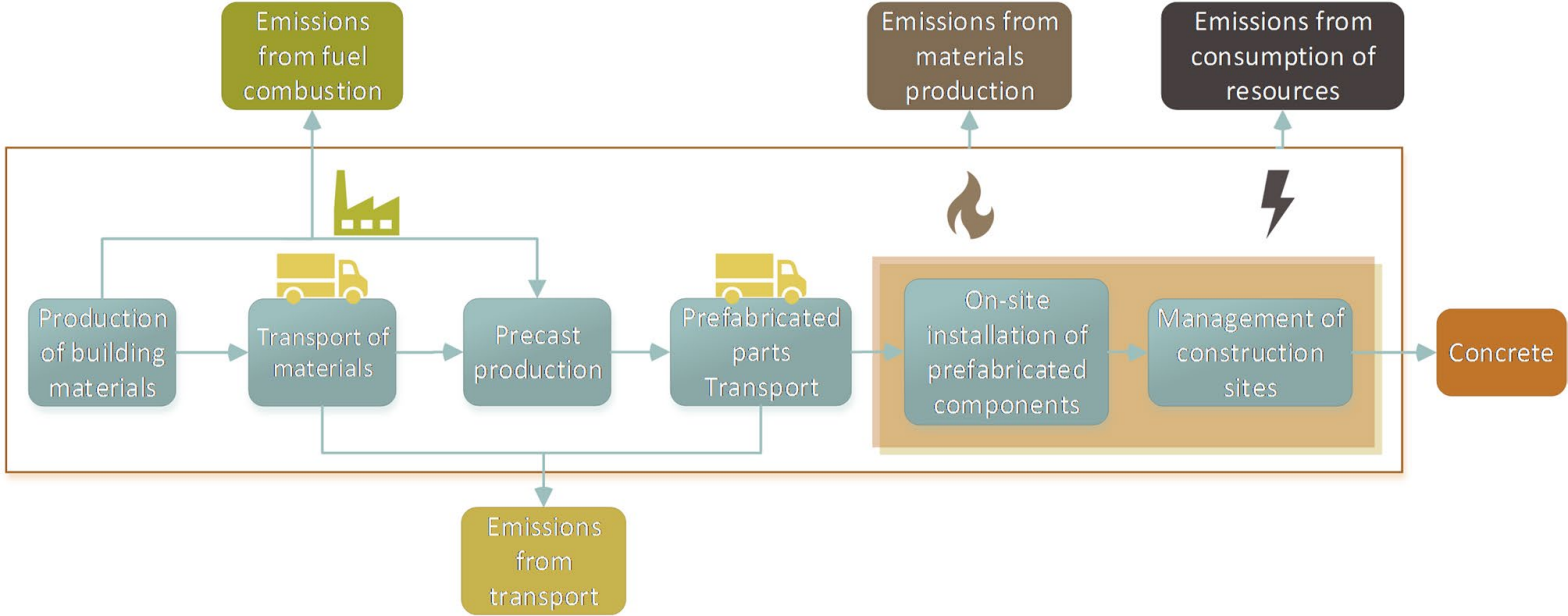
Carbon accounting boundaries for concrete projects.

In Eq. (7), $\:{E}_{concrete}$ represents the total carbon emissions of a concrete project, $\:{E}_{fuel}$ represents the carbon emissions from fuel combustion in the concrete project, $\:{E}_{produce}$ represents the carbon emissions from the production process of raw materials, $\:{E}_{EW}$ represents the indirect carbon emissions from the consumption of intermediate links such as electricity and water resources, and $\:{E}_{transport}$ represents the carbon emissions from transport. According to the ISO14064 series of standards, the boundary of carbon emission accounting for concrete projects is defined as shown in Fig. 2.

Carbon emissions from fuel combustion can be calculated using Eq. (4). Carbon emissions from the feedstock production stage can be calculated using Eq. (8):

8 $$\:{E}_{produce}={\sum\:}_{i=1}^{n}{AD}_{i}\times\:{EF}_{i}$$

In Eq. (8), is the consumption of feedstock i and is the carbon emission factor of feedstock i.

Carbon emissions during construction can be calculated using Eq. (9):

9 $$\:{E}_{EW}=\sum\:_{i=1}^{n}A{D}_{i}\times\:E{F}_{i}$$

In Eq. (9), since the indirect emission source of the concrete project is mainly the consumption of electric energy, $\:A{D}_{i}$ mainly refers to the consumption of electric energy, and $\:E{F}_{i}$ is its corresponding carbon emission factor. Carbon emissions from the raw material transport stage can be calculated by Eq. (10):

10 $$\:{E}_{transport}=\sum\:_{i=1}^{n}A{D}_{i}\times\:{D}_{i}\times\:{T}_{i}$$

In Eq. (10), $\:{D}_{i}$ is the average transport distance of feedstock i in km, and $\:{T}_{i}$ is the carbon emission factor of feedstock i per unit mass and per unit transport distance.

Calculation of carbon emissions from aluminium electrolysis companies.

**Fig. 3:**
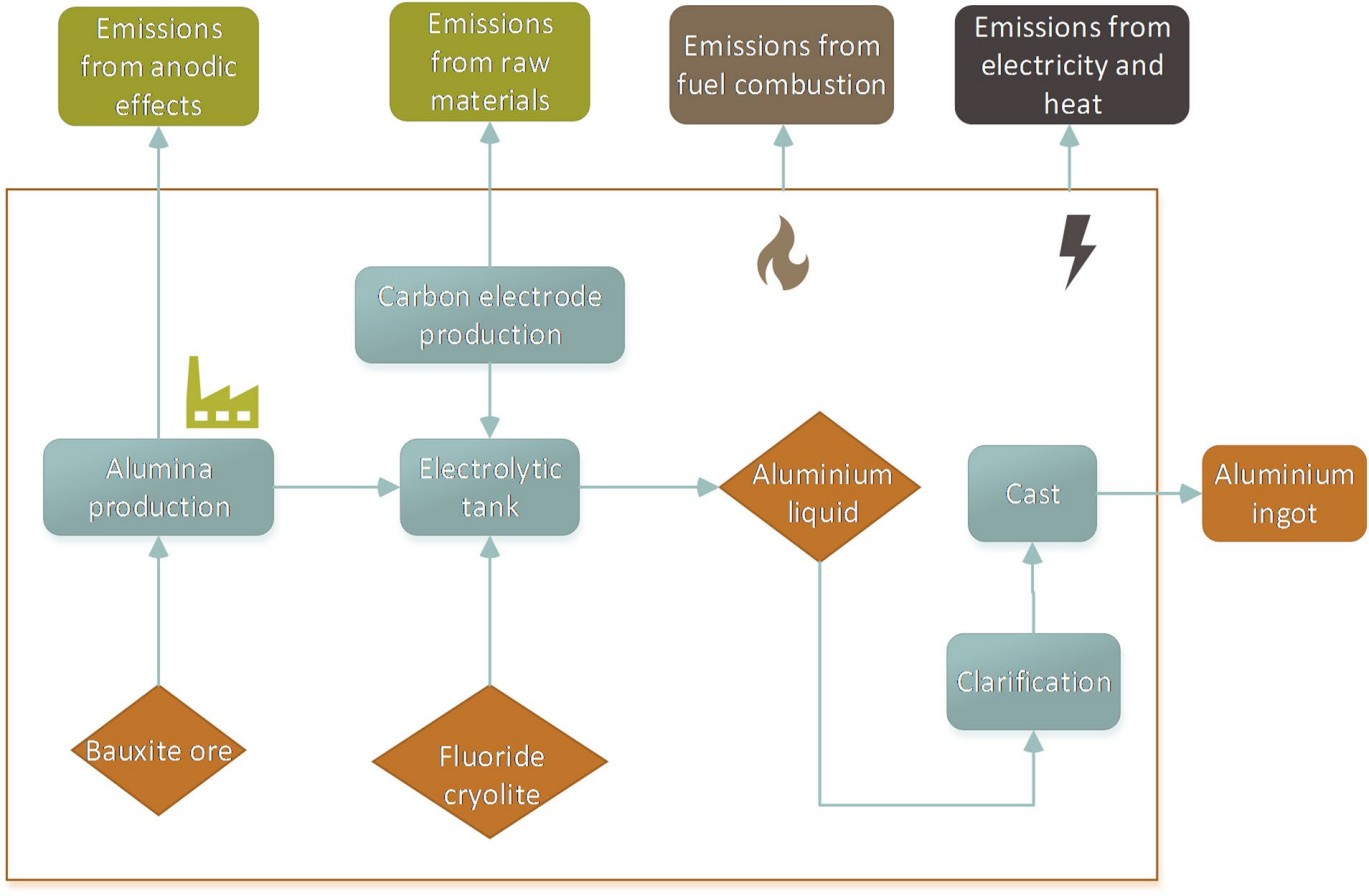
Carbon emission accounting boundaries for electrolytic aluminum companies.

In addition to fuel combustion emissions, this paper summarises the process emissions of aluminium electrolysis enterprises as emissions from the use of energy as a raw material (carbon dioxide emitted during the production of alumina, the raw material used for aluminium electrolysis, in the process of aluminium production) and carbon emissions from the anode effect, and the indirect emissions including those from purchased electricity and heat, with electricity predominating. The total carbon emissions of aluminium electrolysis enterprises can be expressed by Eq. (11):

11 $$\:{E}_{aluminium}={E}_{Fuel}+{E}_{produce}+{E}_{EH}$$

In Eq. (11), $\:{E}_{aluminium}$ represents the total carbon emissions of an aluminium electrolysis enterprise, $\:{E}_{Fuel}$ represents the carbon emissions from fuel combustion in an aluminium electrolysis enterprise,$\:{E}_{produce}$ represents the carbon emissions from the production process of aluminium electrolysis in the process of production, including the net anode consumption of raw materials and emissions from the anode effect in the process, and $\:{E}_{EH}$ represents the indirect carbon emissions from the consumption of electric power and heat, all of which are measured in tonnes.

According to the ISO14064 series of standards, the boundary of carbon emission accounting for electrolytic aluminium enterprises is defined as shown in Fig. 3.

Carbon emissions from fuel combustion can be calculated using Eq. (4). In the estimation of carbon emissions from the production process of electrolytic aluminium, there is Eq. (12):

12 $$\:{E}_{produce}={E}_{Material}+{E}_{anodic}$$

Carbon emissions $\:{E}_{Material}$ from net anode consumption for the production of electrolytic aluminium can be calculated using Eq. (13):

13 $$\:{E}_{produce}=P\times\:{EF}_{i}$$

In Eq. (13), $\:P$ is the total activity level of the firm, i.e., aluminium production, and $\:{EF}_{i}$ is the carbon emission factor for carbon anode consumption.

## The TCN-Transformer model based carbon emission prediction framework

**Fig. 4:**
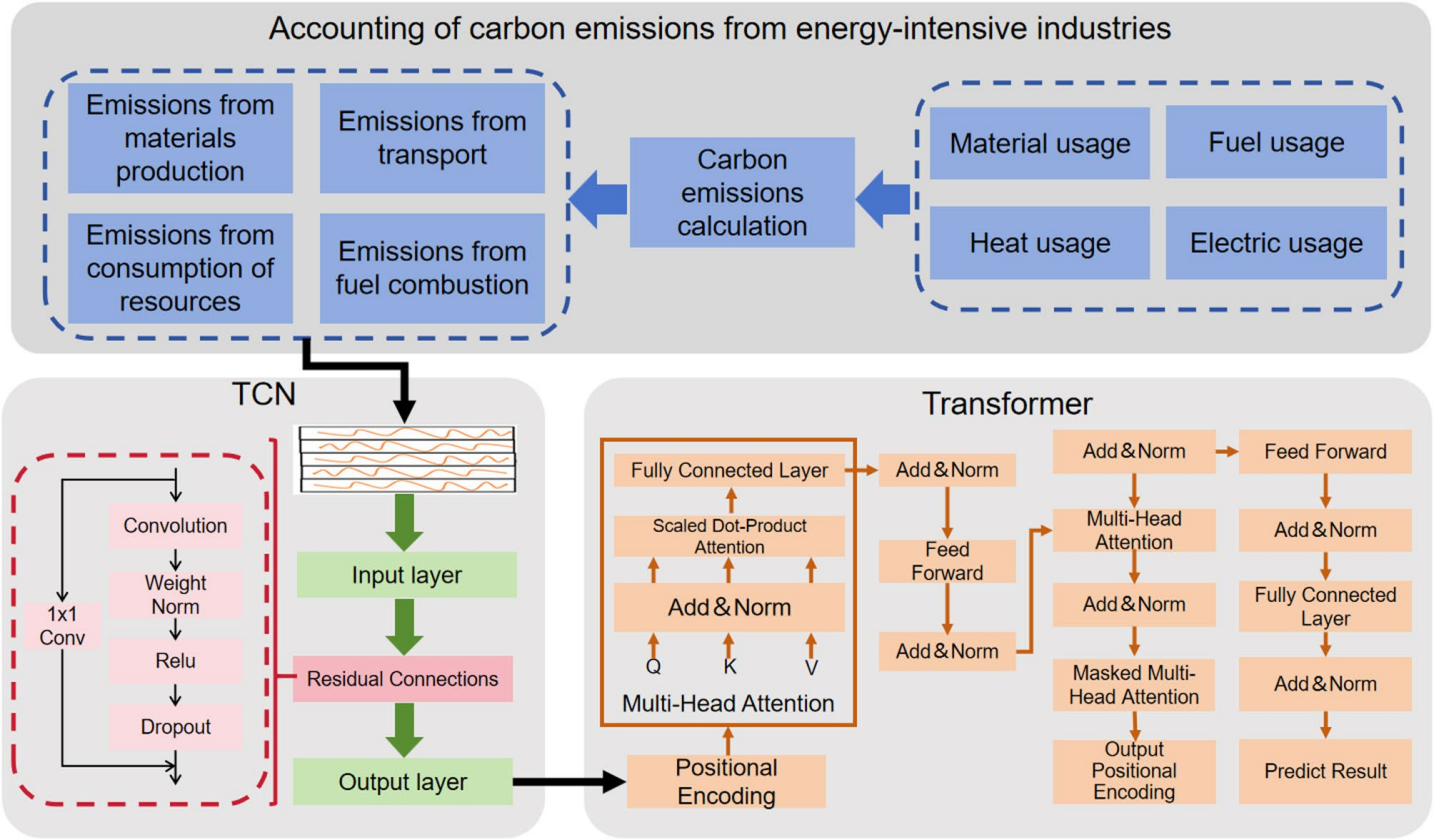
Framework for carbon emission accounting and TCN-Transformer-based prediction.

Figure 4 shows the overall method used in this study. First, the carbon emissions of steel, concrete, and electrolytic aluminum enterprises are calculated based on fuel consumption, raw material use, electricity and heat usage, following standard emission factors. These historical data are then used as input for the TCN-Transformer model, which combines TCN’s ability to extract multi-scale time features with the Transformer’s attention mechanism for learning long-term patterns. This combined model improves the accuracy and reliability of carbon emission forecasting for energy-intensive industries. The input tensor has a shape of [batch_size, lookback_window = 12, num_features = 6], where the lookback window covers the past 12 months. The 12-month setting was chosen to reflect the monthly resolution of the dataset and to capture potential annual periodicity and seasonal variations in industrial production and carbon emission patterns. Since energy-intensive industrial enterprises often exhibit recurring yearly operating characteristics influenced by production schedules, climate conditions, and energy consumption cycles, using a 12-month historical window helps the model retain a complete annual context for short-term forecasting. The six time-varying input features used in the model are fuel consumption, electricity consumption, heat consumption, raw material consumption, carbon anode consumption, and transport distance of raw materials. The input features are shown in Table 1.

**Table 1:** Input Features of the TCN-Transformer Model.

Feature Name	Unit	Data Source
Fuel Consumption	Tons (t) or Kilograms (kg)	Enterprise energy use records, energy metering systems, ISO 14,064 statistics
Electricity Consumption	Kilowatt-hours (kWh)	Enterprise power monitoring systems, electricity billing records
Heat Consumption	Gigajoules (GJ) or kWh	Thermal energy usage logs, energy settlement data
Raw Material Consumption	Tons (t)	Process operation records, raw material inventory and input/output logs
Carbon Anode Consumption	Tons (t)	Production logs of electrolytic aluminum enterprises, anode replacement records
Transport Distance of Raw Materials	Kilometers (km)	Enterprise logistics records, transportation contracts or procurement data

### TCN-based pattern capture mechanism

The Temporal Convolutional Network (TCN), proposed in 2016, has demonstrated strong performance in sequence modeling and time series forecasting tasks due to its efficient architecture and ability to capture long-term dependencies. Unlike traditional recurrent neural networks, TCN utilizes one-dimensional causal convolution, dilated convolution, and residual connections, enabling fewer parameters, better parallelism, and higher computational efficiency^15^. Causal convolution ensures that the output at each time step is influenced only by the current and previous inputs, preserving the temporal causality of the data. To overcome the limited receptive field of standard convolution, TCN employs dilated convolution, which introduces gaps between kernel elements to exponentially expand the receptive field without increasing the kernel size. This allows the model to capture long-range dependencies more effectively across layers. Additionally, residual blocks are used to enhance training stability, with each block composed of two convolutional layers followed by nonlinear activation, and equipped with Weight Normalization and Dropout for regularization. These combined mechanisms enable TCN to model complex temporal patterns with both accuracy and robustness.

**Fig. 5:**
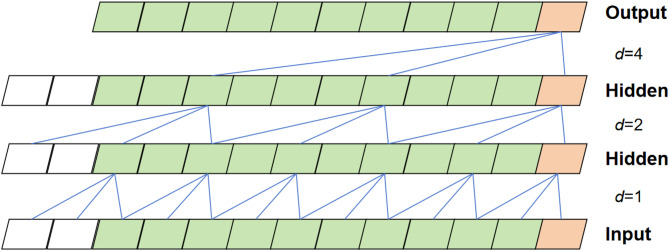
TCN one-dimensional extended causal convolutional structure.

The formula for the expansion convolution is shown in Eq. (14):

14 $$\:F\left(s\right)=\left(x*df\right)\left(s\right)={\sum\:}_{i=0}^{k-1}f\left(i\right)*{x}_{s-d*i}$$

where $\:d$ is the expansion coefficient, $\:k$ is the filter size, and $\:{x}_{s-d*i}$ denotes the convolution of past states. Thus, the inflationary convolution significantly reduces the network complexity and improves the computational efficiency, as shown in Fig. 5.

### Transformer-based self-attention mechanism

**Fig. 6:**
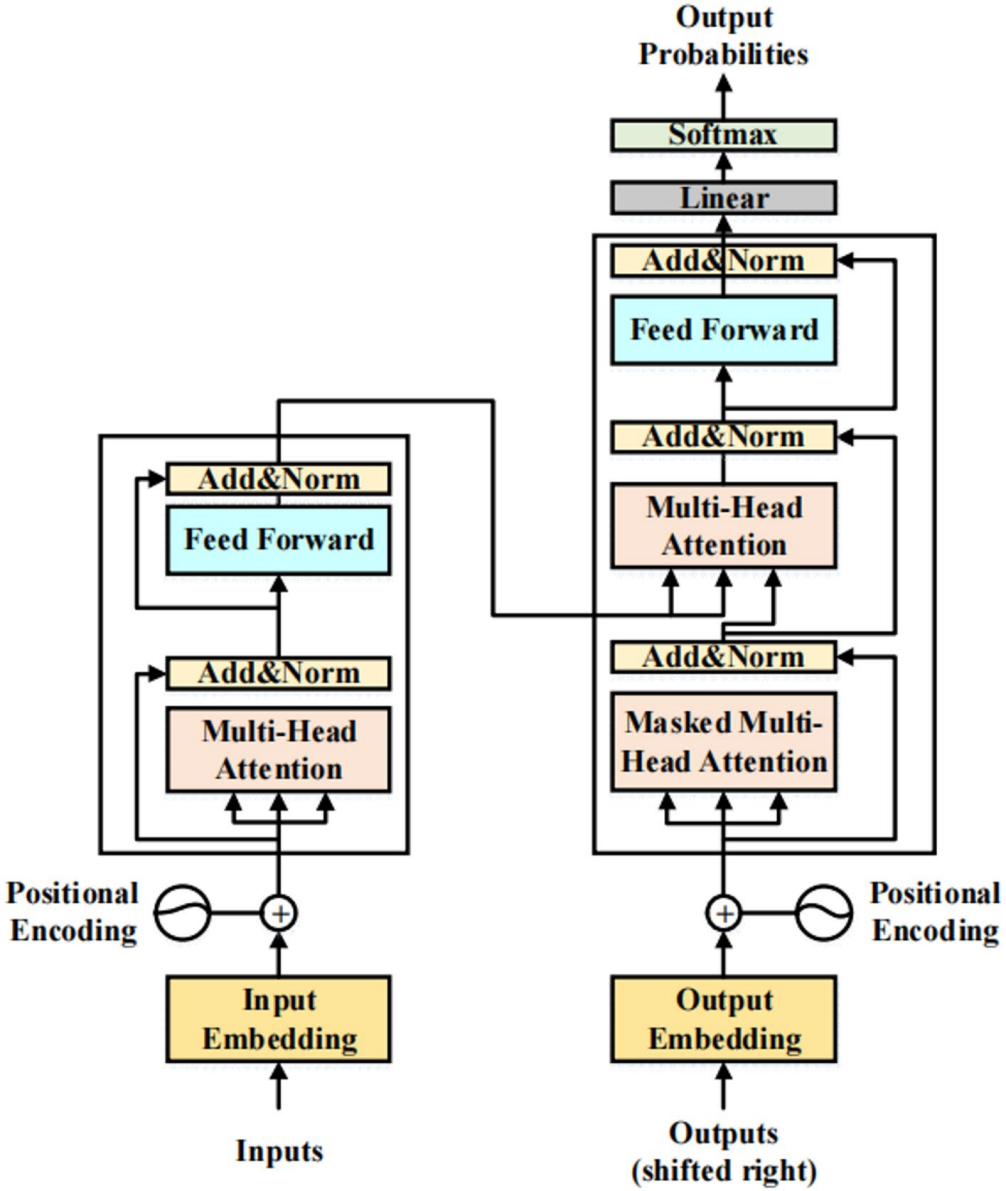
Transformer general structure.

The Transformer architecture consists of two primary components: the Encoder and the Decoder. The Encoder comprises N identical layers, where the input sequence first passes through a Multi-Head Attention mechanism, followed by a residual connection and layer normalization after each operation. The Decoder operates sequentially and includes four key stages: (1) data embedding and positional encoding, where input sequences are embedded and positional information is incorporated to preserve the order of elements; (2) information transfer from Encoder to Decoder, where encoded features guide the decoding process, with masked attention applied to prevent access to future positions and ensure causality; (3) multi-head attention interaction, where the first attention block in the Decoder uses masking, and the second attention block derives key and value matrices from the Encoder’s output, while the query comes from the previous Decoder layer; (4) a Softmax output layer that generates the final prediction results. The attention mechanism is central to Transformer performance, as it enables the model to establish dependencies between any two positions in a sequence by assigning higher weights to more relevant time steps, thereby outperforming traditional time series methods in capturing long-range and context-aware relationships^16,^^17^. Furthermore, residual connections are essential for maintaining gradient flow in deep architectures and preserving original input information. Without them, the output can become detached from its source due to the random initialization of query and key vectors, resulting in degraded performance. Lastly, layer normalization plays a vital role in ensuring stable and efficient training, while the integrated feed-forward network in each block enables independent transformation of each sequence element, thus enhancing the model’s overall representation capacity, as shown in Fig. 6.

The transformer’s self-attention mechanism can be formulated as Eq. (15):

15 $$\:A(K,Q,V)=S\left(\frac{Q{K}^{T}}{\sqrt{{d}_{k}}}\right)V$$

where $\:A$ represents the attention mechanism; $\:S$ represents the Softmax function that calculates the attention weights; $\:K,Q,V$ calculates the matrix of the attention mechanism, the keys of the matrix and the values, respectively; $\:{d}_{k}$ is the dimension of the keys.

The multiple attention mechanism can be expressed as Eq. (16):

16 $$\:\begin{array}{c}{M}_{h}=C({h}_{1},{h}_{2},{h}_{3},L,{h}_{i}){W}_{o}\\\:{h}_{i}=A\left(Q{W}_{Qi},Q{W}_{Ki},Q{W}_{Vi}\right)\end{array}$$

where $\:{M}_{h}$ is the multi-head attention mechanism; $\:C$ is the connection mechanism between the attention; $\:{h}_{i}$ is denoted as the i-th attention mechanism; $\:{W}_{o}$ is the linear transformation weight matrix after the connection of the multi-head attention mechanism; $\:{W}_{Qi}$,$\:{W}_{Ki}$,$\:{W}_{Vi}$ are the linear transformation weight matrix.

**Fig. 7:**
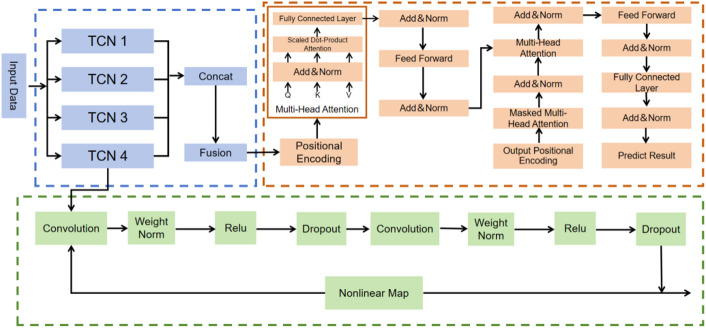
Network timing prediction process of TCN-Transformer.

### TCN-Transformer joint prediction mechanism

In this study, a hybrid TCN-Transformer deep learning model is employed for time series prediction. The model integrates a TCN module for multi-scale temporal feature extraction and a Transformer module for parallel attention-based sequence modeling. The TCN component utilizes dilated convolutions to expand the receptive field and capture features at various temporal scales, while residual connections enhance gradient flow, enabling deeper temporal representation learning. In Fig. 7, the green module represents the Temporal Convolutional Network (TCN), which is responsible for extracting multi-scale temporal features from the input time series. These features are then passed through a linear transformation layer to align the dimensions with the Transformer encoder’s input. The output of the Transformer decoder is then mapped to final predictions through a fully connected layer. This modular pipeline ensures both local pattern recognition and long-range dependency modeling. The extracted features are then passed to the Transformer, where positional encoding is applied to preserve temporal order, followed by multi-head attention to learn dependencies across time steps. To prevent future information leakage, the Transformer decoder incorporates masked attention, and an additional attention layer focuses on encoder outputs. The final predictions are generated through a fully connected layer and a linear activation function, which is standard for regression tasks such as carbon emission forecasting, ensuring that output values remain in a continuous domain, as illustrated in Fig. 7.

## Case studies

In this study, a TCN-Transformer model was constructed using MATLAB to perform time series forecasting of annual carbon emissions for steel enterprises, concrete enterprises, and electrolytic aluminum enterprises. The model incorporates both temporal convolution and attention mechanisms to improve the accuracy and robustness of sequential prediction. Key hyperparameters of the model include TCN input dimension, number of TCN layers, dilation factors, TCN output dimension, Transformer embedding dimension, hidden layer size, number of attention heads, the number of encoder-decoder stacks, and the number of training epochs, as shown in Table 2.

The dataset was collected from field investigations of three representative energy-intensive enterprises located in northern Henan Province, China, including a steel plant, a cement plant, and an electrolytic aluminum plant. These enterprises were selected because they represent typical high-energy-consuming industrial sectors with distinct production processes and carbon emission characteristics. The data were obtained through factory surveys and on-site investigations, including enterprise energy consumption records, production-related activity data, and carbon-emission-related statistical information.

**Table 2:** TCN-Transformer parameters.

Parameter	Value	Parameter	Value
TCN Input Dimension	6	Number of Heads of Attention	8
TCN Network Layers	4	Number of encoder-decoder stacks	2
Expansion Factor	[1, 2, 4, 8]	Maximum/minimum learning rate	1e-3/5e-5
TCN Output Dimension	64	Epoch	100
Transformer Embedding Dimension	64	Transformer Hidden Layer	64
Optimizer	Adam	Batch size	64
Learning Rate Schedule	Noam Scheduler	Early Stopping	10

Prior to model training, all input features were normalized using Min-Max scaling to the range [0,1], based on the training dataset statistics. Missing values, which accounted for less than 2% of total records, were filled using linear interpolation. The full dataset was split into training (80%) and testing (20%) sets following chronological order, ensuring no data leakage across the temporal boundary. The total receptive field of the TCN component is calculated as 31 time steps, given 4 layers with kernel size 3 and dilation rates [1, 2, 4, 8]. Since the input data is monthly carbon emission records, this receptive field spans over 2.5 years, which sufficiently captures annual and seasonal cycles inherent in industrial carbon emissions.

To avoid ambiguity, we clarify that all results reported in the revised manuscript were obtained using the corrected TCN-Transformer architecture with a linear output layer for regression. After identifying that a Softmax-based output formulation was inappropriate for continuous carbon emission prediction, we re-ran all experiments, including model training, testing, and metric evaluation, under the corrected regression setting. Therefore, all figures, tables, and performance metrics presented in the current manuscript correspond to the corrected model implementation.

**Fig. 8:**
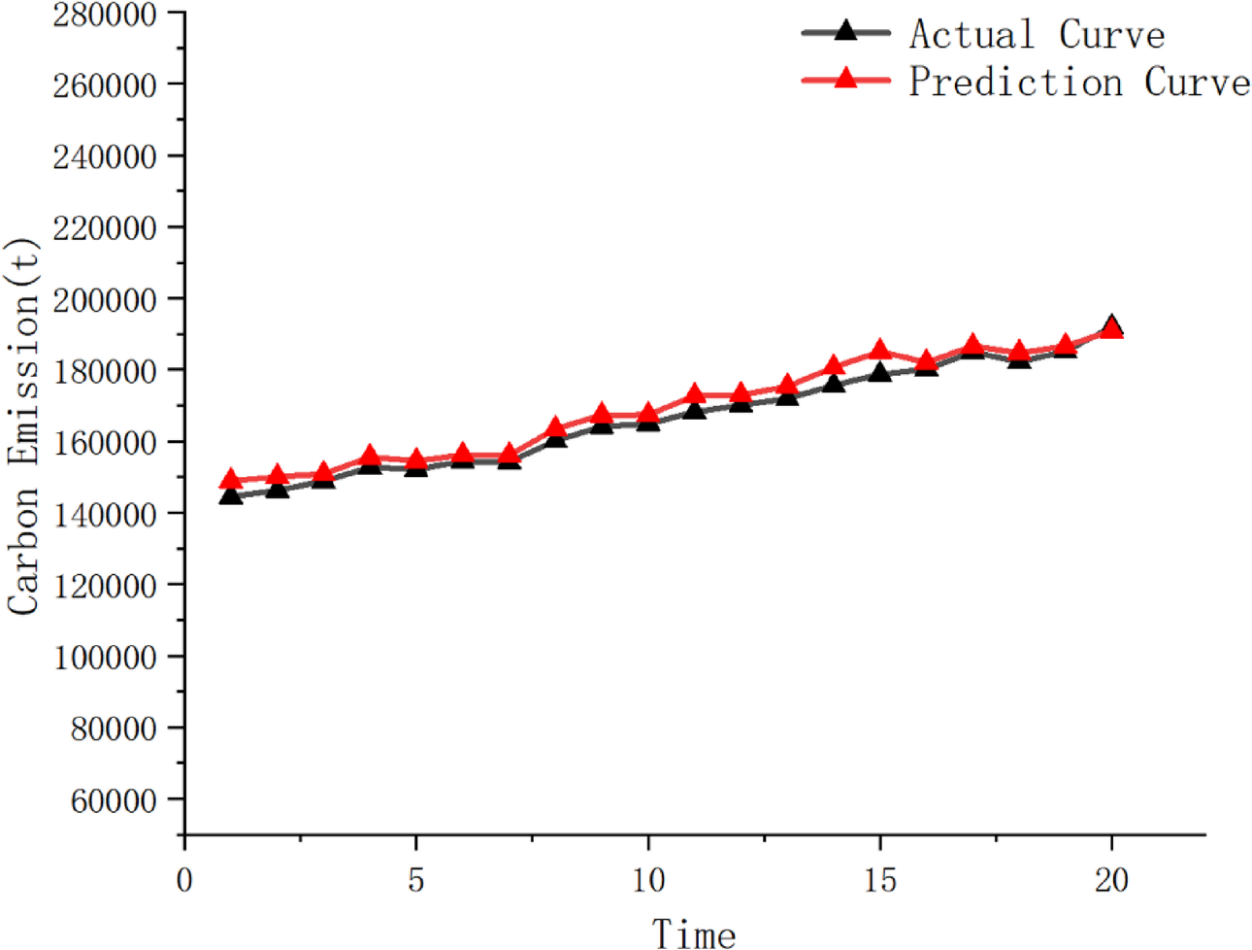
Carbon emission forecasts for steel companies.

**Fig. 9:**
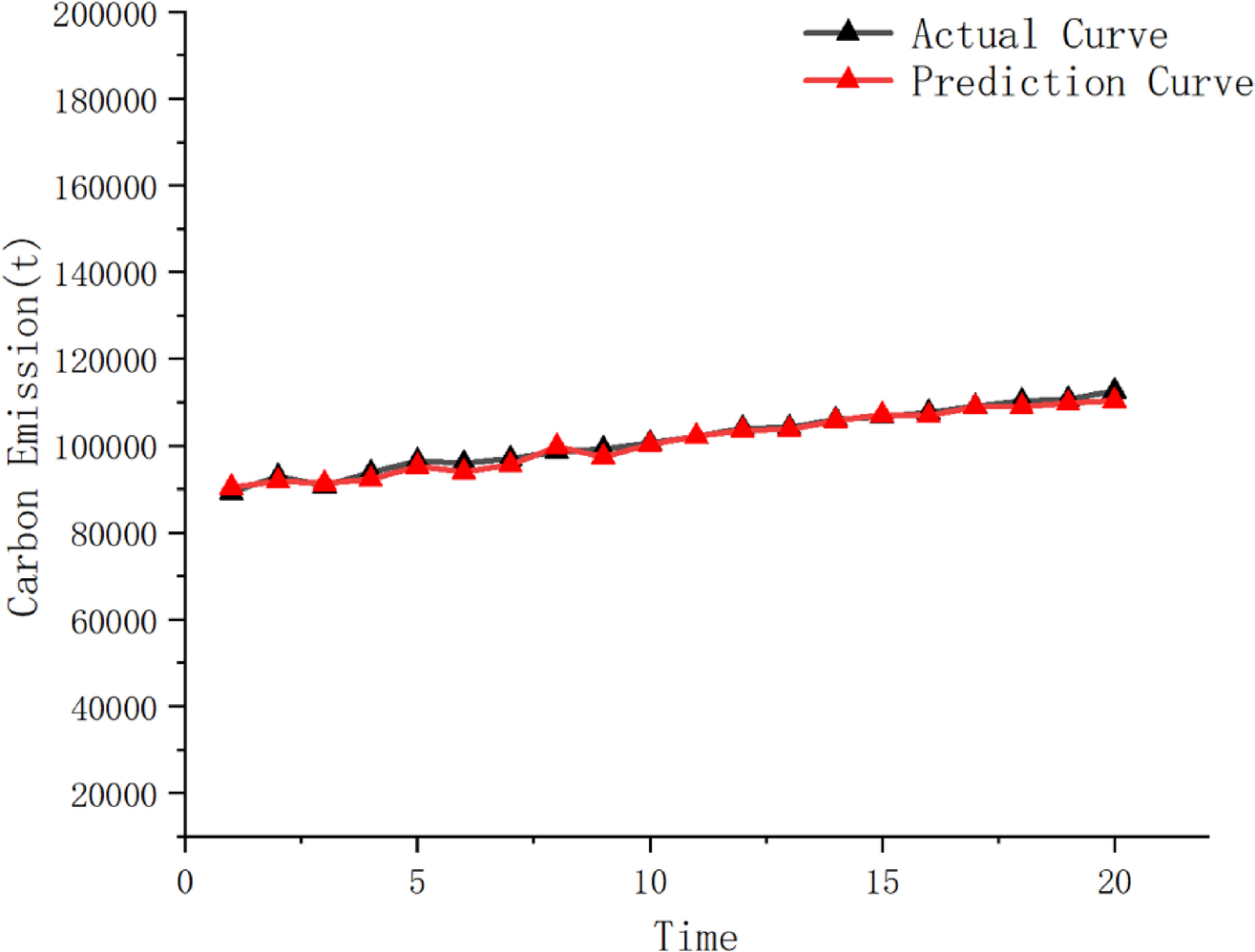
Carbon emission projections for concrete companies.

**Fig. 10:**
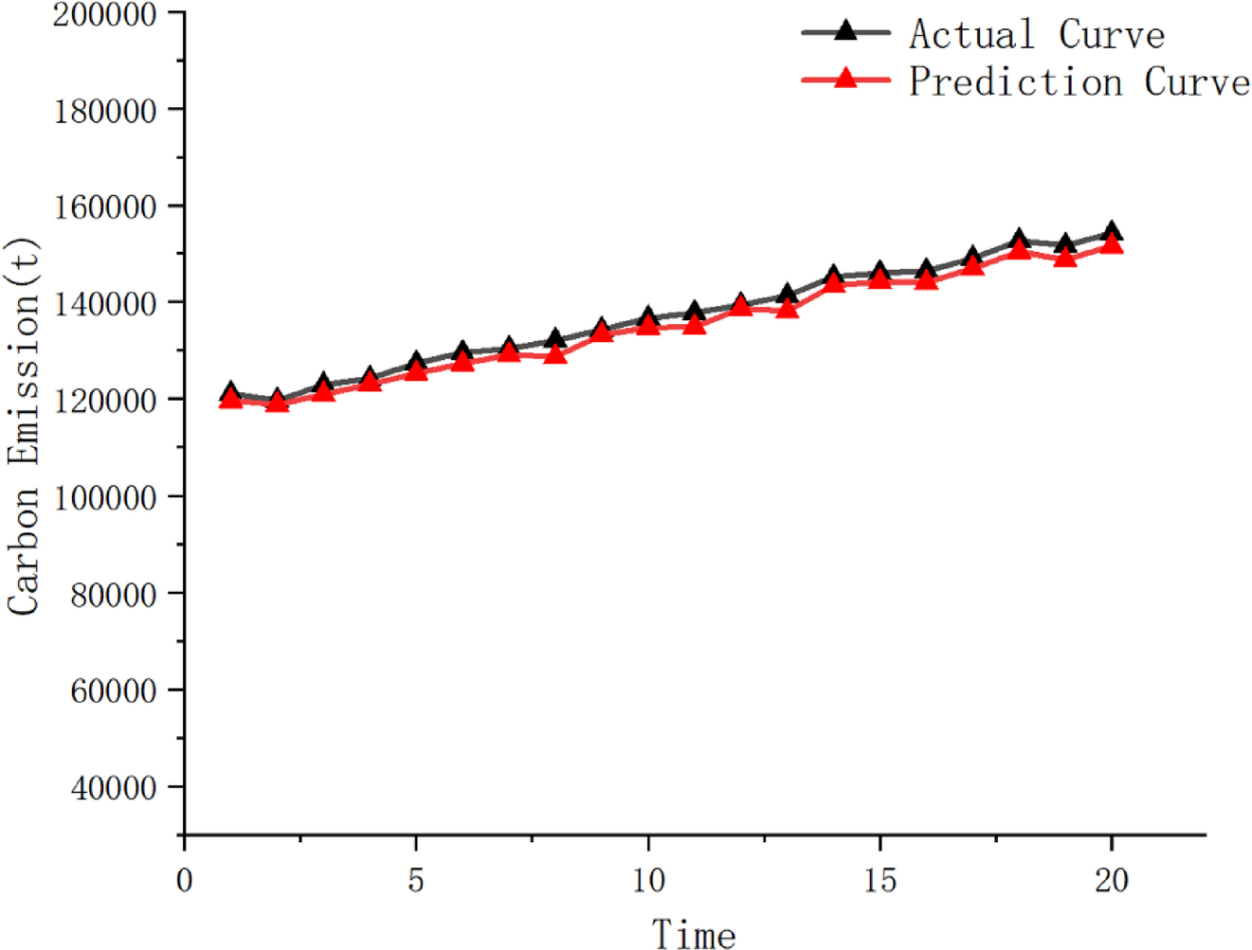
Carbon emission projections for aluminum electrolysis companies.

Line plots comparing historical and predicted carbon emissions for the three enterprise types are shown in Figs. 8, 9 and 10. These plots demonstrate that the TCN-Transformer model effectively captures the overall trend of emissions across all three sectors, with predicted values closely aligning with historical observations. The carbon emission data employed in this study, which span a 20-year with monthly resolution, were collected from three representative energy-intensive enterprises in China. For details, please refer to Supplementary Table 1: Relevant Consumption and Carbon Emission Data of Three Enterprises (Aluminum Electrolysis Company, Concrete Company, Steel Company).

To ensure the interpretability and fairness of the evaluation, we compared the proposed method with the Transformer model, which serves as a strong baseline in time-series forecasting tasks. We adopted a train-test split validation protocol, in which 80% of the data was used for training and the remaining 20% for testing. The dataset consists of monthly records spanning a period of 20 years with monthly resolution, providing sufficient data to support robust model evaluation.

**Fig. 11:**
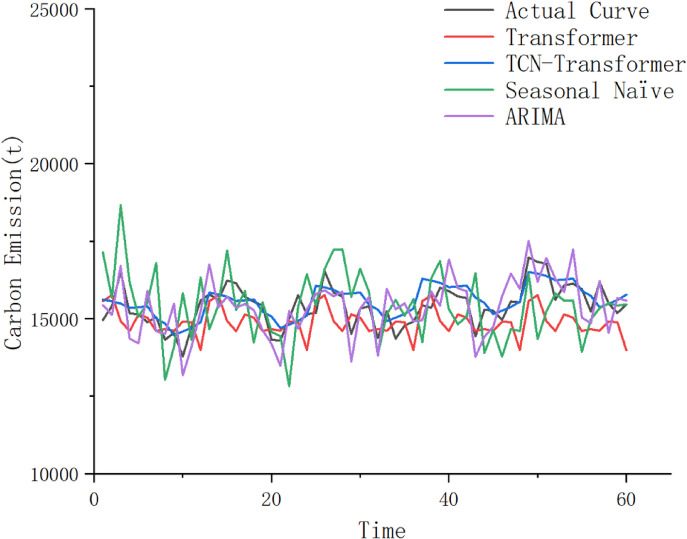
Comparison of prediction results of various algorithms.

All prediction results presented in Figs. 8, 9, 10 and 11 were obtained using a lookback window of 12 months and a one-month forecast horizon, with the evaluation period covering the final 20% of the dataset.

Figure 11 presents a direct comparison between the standalone Transformer model and the proposed TCN-Transformer model using monthly carbon emission data from an electrolytic aluminum enterprise. Since the key structural difference between these two models is the introduction of the TCN module, this comparison also serves as a direct validation of the effectiveness of the proposed hybrid architecture. Although both models are capable of capturing temporal dependencies, the TCN-Transformer achieves better prediction performance by combining the Transformer’s long-range dependency modeling capability with the TCN’s strength in local and multi-scale temporal feature extraction. In particular, the TCN module enhances the model’s ability to identify subtle short-term fluctuations and local temporal patterns that may not be fully captured by self-attention alone. As shown in Fig. 11; Table 3, the proposed TCN-Transformer consistently outperforms the standalone Transformer, which confirms that the introduced hybrid structure contributes meaningfully to the observed accuracy improvement. To further contextualize the predictive performance of the proposed method, we also compare it with widely used statistical baselines, including Seasonal Naïve and ARIMA. These baselines provide interpretable references and help demonstrate the advantage of the proposed deep hybrid model in industrial carbon emission forecasting.

**Table 3:** Performance comparison of prediction models.

Method	RMSE (± SD)	MAE (± SD)	MAPE (± SD)	FSI
Seasonal Naïve	1124.65 ± 61.52	975.31 ± 48.23	7.21% ± 0.65%	0.000
ARIMA	869.43 ± 52.18	763.44 ± 38.57	5.73% ± 0.44%	0.227
Transformer	787.84 ± 47.27	687.10 ± 39.23	4.99% ± 0.52%	0.299
TCN-Transformer	437.67 ± 26.26	360.49 ± 21.63	3.21% ± 0.29%	0.610

In addition to traditional error metrics (RMSE, MAE, MAPE), we also report the Forecast Skill Index (FSI), a relative performance measure against the Naïve baseline. FSI provides a normalized view of forecast accuracy improvements and helps gauge the true value of model complexity.

To further contextualize the prediction accuracy of the proposed TCN-Transformer model, we compared its MAPE with values reported in existing literature. For instance, Guo et al^8^. reported a MAPE of 5.1% using TCN-BiLSTM-AM for residential load forecasting, and Zhao et al^9^. achieved 4.7% MAPE using TCNMS-BiLSTM. In contrast, our model attained a MAPE of 3.21% on real-world monthly carbon emission data from electrolytic aluminum enterprises, demonstrating a substantial improvement. This highlights the proposed method’s capacity to capture complex emission dynamics with higher temporal fidelity, particularly in industrial contexts where data heterogeneity and periodicity pose modeling challenges.

## Conclusion

This study demonstrates the effectiveness of the proposed TCN-Transformer model in forecasting carbon emissions for energy-intensive industries. By integrating the feature extraction capabilities of TCN and the sequence modeling strengths of the Transformer, the model achieves high prediction accuracy across steel, concrete, and electrolytic aluminum sectors. Comparative analysis indicates that the TCN-Transformer outperforms standalone Transformer models by capturing both local and long-range temporal dependencies. The use of real enterprise-level data ensures the practical relevance of the results. This approach provides a reliable tool for carbon emission trend prediction and can support emission reduction strategy development and industrial carbon audits.

Despite the improved performance demonstrated by the TCN-Transformer model, several limitations should be acknowledged. First, the model primarily relies on historical carbon emission data and does not incorporate real-time production or policy-related variables, which may affect prediction robustness in rapidly changing contexts. Second, the model assumes the availability of high-quality, continuous time-series data, which may not hold in all industrial scenarios. Lastly, while the model performs well for monthly prediction tasks, its effectiveness for finer time resolutions (e.g., daily or hourly forecasting) remains to be validated. These limitations suggest promising directions for future work, such as integrating exogenous features and adapting the model for multi-resolution forecasting tasks.

Future work will explore the extension of this framework to multi-step forecasting scenarios, enabling longer-term emission trend analysis. Additionally, the incorporation of exogenous variables such as production activity levels, weather conditions, and policy shifts will be investigated to further enhance the model’s predictive power and applicability to real-world decision-making contexts. This progression will support more comprehensive and adaptive carbon management strategies for industrial sectors under dynamic environmental and regulatory conditions.

## Supplementary Information

Below is the link to the electronic supplementary material.

Supplementary Material 1

## Data Availability

All data is available upon request. Please contact B.Z. (bw.zheng@outlook.com) if someone wants to request the data from this study.
